# Editorial: Untapped metabolic diversity in legume-characteristic pathways

**DOI:** 10.3389/fpls.2022.1117833

**Published:** 2023-01-10

**Authors:** Mehran Dastmalchi, Sangeeta Dhaubhadel

**Affiliations:** ^1^ Plant Science, McGill University, Sainte-Anne-de-Bellevue, QC, Canada; ^2^ London Research and Development Centre, Agriculture and Agri-Food Canada, London, ON, Canada; ^3^ Department of Biology, University of Western Ontario, London, ON, Canada

**Keywords:** legumes, transcriptomics, metabolomics, cytochrome P450 (CYP), phytoalexins, specialized metabolism

In this special issue, new insights are found into the specialized metabolism of legumes and the gene families that play a significant role in defence and stress response. Plant defence is strictly regulated to allocate resources when and where needed, including an arsenal of constitutively expressed *first responders* and bioactive derivatives (*phytoalexins*) deployed *de novo* (VanEtten et al., 1994). From the perception of stress, damage, or infection, an intricate signalling cascade is triggered, *via* phytohormones, rendering changes, including cell wall modifications (Lionetti et al., 2007), metabolic shifts (Simons et al., 2011; Sukumaran et al., 2018), and the biosynthesis of specialized defence compounds (Förster et al., 2022).

Researchers in this special issue have probed the underlying molecular factors responding to stresses, including salt and mercury stress (Wang et al.; Alvarez-Rivera et al.) and *Phytophthora sojae* infection (Khatri et al.), as well as transport mechanisms for sequestration of specialized metabolites (Islam et al.). They have also developed metabolo-transcriptomic databases to investigate medicinal plants (Lin et al.) and leveraged these pathways *via* plant/heterologous culture bioreactors (Istiandari et al.; Lee et al.). Deep understanding of this metabolic “design space,” using the resources reported in this issue, can help us tap into the potential of plant specialized metabolism and eventually move beyond the plant as a host or its catalogue of compounds.

Two papers in this issue discuss the merits of the medicinal legume *Glycyrrhiza uralensis* or Chinese licorice (Istiandari et al.; Wang et al.). This plant has rhizomes that can be harvested in early autumn, dried, and pounded into a saccharine powder, 50 times sweeter than sugar (*Glykys*, meaning sweet in Greek). This natural demulcent has a soothing effect on the digestive tract. *Glycyrrhiza* species are also a source of myriad legume-characteristic metabolites, notably including glycyrrhizin, a saponin molecule.

Environmental stresses, including salinity, can impact the composition and content of these roots (Behdad et al., 2020). Therefore, Wang et al. used an integrated metabolo-transcriptomic approach to identify differentially expressed genes (DEGs) in *G. uralensis* under salt stress. Over 3,000 DEGs were identified, notably including two cytochrome P450s (CYPs) involved in glycyrrhizin biosynthesis, CYP88D6 and CYP72A154 and two putative UDP glycosyltransferases (UGTs). The latter are candidate UGTs for the decoration and storage of flavonoid glycosides and triterpenoid saponins, which would be expected in the rhizomes of licorice.

In the other paper on *G. uralensis*, and a critical proof-of-concept work, the authors leveraged the metabolic diversity of this species (Istiandari et al.). They compared the efficacy of CYPs with various inter/intra-specific electron-donating partners: the NADPH-cytochrome P450 reductase (CPR). CPRs from classes I and II were characterized in combination with CYPs from several metabolic categories. They deployed a CYP88D6 from *G. uralensis*, which performed best when paired with a class II CPR from the same species, producing 11-oxo-β-amyrin, an essential precursor of the high-value glycyrrhizin. Therefore, Istiandari et al. have highlighted a significant blind spot in engineered bio-chassis work, which overlooks isoform and intra-specific partnerships between CPR and CYP groups.

Elsewhere in the issue, researchers have highlighted the utility of the metabolo-transcriptomic approach in building databases for gene discovery and pathway elucidation in both model and non-model legumes. Alvarez-Rivera et al. profiled *Medicago truncatula* under mercury (Hg) stress response, discovering that a suite of isoflavone aglycones, including biochanin A, daidzein, and irisolidone (5,7-dihydroxy-6,4ʹ-dimethoxyisoflavone), were enriched in the roots. Knowledge of the associated genes and the cocktail of defence compounds can be used to confer Hg stress tolerance to susceptible plants. This arsenal can be expanded by looking to non-model legumes, such as the unique profile produced by the “box bean” or *Entada phaseoloides*, reported by Lin et al. These databases can form the basis of future work characterizing lineage-specific neo/sub-functionalization in flavonoid structural enzymes.

An example of pathogenic stress response was investigated by Khatri et al., comprehensively cataloging all soybean (*Glycine max*) *CYPs* upregulated in response to *Phytophthora sojae* infection (a soil-borne oomycete pathogen responsible for soybean root rot). They identified three candidate members of the CYP71 family with over 10-fold upregulation upon infection. Khatri et al. speculate that these *CYPs* could be actively transcribed to increase flux into glyceollin biosynthesis, a phytoalexin pterocarpan characteristic of soybean roots.

While specialized metabolites can provide a layer of protection to plant tissues, they can also detract from the commercial value of agricultural products (Elango et al.). Such is the case with proanthocyanidins (PAs), which are deposited in the endothelial layer of the seed coats, creating a pre-formed *first-response* protective barrier. Nevertheless, postharvest seed darkening in common bean (*Phaseolus vulgaris*) varieties, such as pinto beans (Park and Maga, 1999), leads to substantial economic losses. Mechanistically, PA monomers are synthesized in the cytosol and transported by a tonoplast transporter to the vacuole for polymerization. Islam et al. identified a multidrug and toxic compound extrusion (MATE) transporter, PvMATE8, that transports the PA monomer, epicatechin 3ʹ-O-glucoside, into the vacuole. This work follows up on previous research that pinpointed the responsible locus for the “slow darkening trait” in pinto beans, providing an essential molecular marker for the breeding community (Islam et al., 2020).

Finally, in a technological paper, Lee et al. used soybean adventitious root (AR) cultivation at laboratory (3 L) and pilot (1,000 L) scale bioreactors to produce high-value legume phytoalexins. This venture revealed the promise and limitations of such an approach. In constant light and methyl jasmonate (phytohormone) treatment, the AR cultures produced (malonyl)glycosylated derivatives of coumestrol. Such metabolites can be used in the treatment of hormone-dependent cancers. However, the bioreactors often yield isomeric mixtures and complex cocktails, which require costly purification.

This special issue on legume-characteristic specialized metabolism highlights the importance of the scientific approach to tackling metabolic pathways within the context of plant speciation and architecture. Leveraging these tools for a mechanistic understanding of the molecular factors is the next and, perhaps, more challenging hurdle, but we can be reassured by the breadth and depth of resources. Knowledge of the lattice of lineage-specific pathways will provide value for agrochemical, nutritive and medicinal compound bioengineering, whether *in situ* or in bioreactors.

## Author contributions

All authors listed have made a substantial, direct, and intellectual contribution to the work and approved it for publication.

## References

[B1] Behdad A. Mohsenzadeh S. Azizi M. Moshtaghi N. (2020). Salinity effects on physiological and phytochemical characteristics and gene expression of two glycyrrhiza glabra l. populations. Phytochemistry 171, 1–10. doi: 10.1016/J.PHYTOCHEM.2019.112236 31923723

[B2] Förster C. Handrick V. Ding Y. Nakamura Y. Paetz C. Schneider B.. (2022). Biosynthesis and antifungal activity of fungus-induced O-methylated flavonoids in maize. Plant Physiol. 188, 167–190. doi: 10.1093/PLPHYS/KIAB496 34718797 PMC8774720

[B3] Islam N. S. Bett K. E. Pauls K. P. Marsolais F. Dhaubhadel S. (2020). Postharvest seed coat darkening in pinto bean (Phaseolus vulgaris) is regulated by psd, an allele of the basic helix-loop-helix transcription factor p. Plants People Planet 2, 663–677. doi: 10.1002/PPP3.10132 34268482 PMC8262261

[B4] Lionetti V. Raiola A. Camardella L. Giovane A. Obel N. Pauly M.. (2007). Overexpression of pectin methylesterase inhibitors in arabidopsis restricts fungal infection by botrytis cinerea. Plant Physiol. 143, 1871–1880. doi: 10.1104/pp.106.090803 17277091 PMC1851811

[B5] Park D. Maga J. A. (1999). Dry bean (Phaseolus vulgaris) color stability as influenced by time and moisture content. J. Food Proc Preserv. 23, 515–522. doi: 10.1111/J.1745-4549.1999.TB00401.X

[B6] Simons R. Vincken J. P. Roidos N. Bovee T. F. H. Van Iersel M. Verbruggen M. A.. (2011). Increasing soy isoflavonoid content and diversity by simultaneous malting and challenging by a fungus to modulate estrogenicity. J. Agric. Food Chem. 59, 6748–6758. doi: 10.1021/jf2010707 21561073

[B7] Sukumaran A. McDowell T. Chen L. Renaud J. Dhaubhadel S. (2018). Isoflavonoid-specific *prenyltransferase* gene family in soybean: GmPT01, a pterocarpan 2-dimethylallyltransferase involved in glyceollin biosynthesis. Plant J. 96, 966–981. doi: 10.1111/tpj.14083 30195273

[B8] VanEtten H. D. Mansfield J. W. Bailey J. A. Farmer E. E. (1994). Two classes of plant antibiotics: Phytoalexins versus “Phytoanticipins.” Plant Cell 6, 1191–1192doi: 10.1105/tpc.6.9.1191 12244269 PMC160512

